# A Novel Recombinant Protein Purification Approach Using Biomolecular Condensates

**DOI:** 10.3390/ijms27135721

**Published:** 2026-06-25

**Authors:** Yawen Fu, Houjin Zhang

**Affiliations:** MOE Key Laboratory of Molecular Biophysics, Department of Biotechnology, College of Life Science and Technology, Huazhong University of Science and Technology, Wuhan 430074, China; m202472930@hust.edu.cn

**Keywords:** LplA, condensates, liquid–liquid phase separation, protein purification, temperature

## Abstract

The lipoate-protein ligase A (LplA) identified in *Escherichia coli* K-12 exhibits structural homomeric oligomerization and reversible lower critical solution temperature (LCST)-type phase separation in vitro. In this study, based on the ability of LplA to form condensates, it was utilized as a temperature-sensitive purification tag in the field of protein purification for the first time, and a novel and convenient one-step purification method was established. A universal vector was developed for the fusion expression of LplA and the target protein. The fusion protein forms condensates upon heating, separating from the solution, and redissolves in buffer at lower temperatures, enabling the purification of the target protein from cell lysates. Through exploration of phase separation temperatures, 30 °C was determined to be the optimal purification temperature. Subsequently, three enzymes of different molecular sizes (lipase EstA, endoglucanase BcsZ, and endoglucanase EglS) demonstrated the versatility of this condensate-based purification method. Furthermore, the specific activity and purification efficiency of the purified enzymes were comparable to those of enzymes purified by conventional affinity chromatography. This research contributes to the introduction of condensates into protein purification applications, offering potential support for the large-scale production and purification of functional proteins.

## 1. Introduction

In recent years, biomolecular condensates have attracted widespread attention as an important form of intracellular organisation [1]. Condensates are typically formed by proteins or nucleic acids through weak multivalent interactions and can exhibit liquid–liquid phase separation (LLPS) properties [2], thereby forming membraneless compartments within cells [3,4]. Unlike traditional stable complexes, biomolecular condensates are characterized by dynamic reversibility and high environmental responsiveness. Their assembly and disassembly processes are often regulated by factors such as temperature, pH, ionic strength, and molecular concentration [5]. Recent studies have shown that certain proteins can undergo reversible aggregation under specific conditions and can be rapidly separated through simple procedures such as centrifugation [6]. Consequently, condensates not only play a vital role in biological processes such as transcriptional regulation, stress responses, and metabolic regulation [7,8,9], but also offer new avenues of research for biological separation and protein purification techniques.

The separation and purification of proteins are of great significance to biological research. Currently, protein purification primarily relies on methods such as affinity chromatography [10], ion-exchange chromatography [11], and size-exclusion chromatography [12]. Among these, affinity chromatography based on His-tags [13], GST-tags [14], and MBP-tags is the most widely used [15]. Although these methods provide high separation efficiency and purification accuracy, they generally require expensive chromatographic resins and equipment, involve complex operational procedures, and require relatively long purification times. Moreover, large-scale production is often associated with high costs, resin inactivation, and limited reusability. In addition, chromatographic purification typically involves multiple buffer exchange and elution steps, which can readily lead to a reduction in the biological activity of the target protein [16]. To overcome the limitations of traditional chromatography techniques, researchers have begun to explore non-chromatographic protein purification strategies, with stimulus-responsive protein tags attracting increasing attention. These tags undergo reversible conformational changes, aggregation, or phase transitions in response to external stimuli (such as temperature, pH, ionic strength, or small-molecule ligands), thereby enabling the separation of target proteins from contaminants [17]. Temperature-responsive purification tags constitute an important branch of this field, with typical examples including the inverse phase transition cycling (ITC) purification system based on elastin-like polypeptide (ELPs) [18]. ELP tags undergo reversible precipitation upon heating and the addition of salt, enabling protein purification without the need for chromatography resin [19]. However, such systems typically require multiple heating–cooling cycles, involve high heating temperatures, and need high salt concentrations to promote precipitation, all of which have a significant impact on the structure and activity of the target protein [20]. Furthermore, ELP tags require an increase in sequence length to improve separation efficiency, which will affect the expression levels and function of the target protein [21]. These limitations have restricted their practical application and widespread adoption. Therefore, the development of a novel temperature-responsive purification tag that offers good reversibility, mild induction conditions, and high separation efficiency is of great significance for establishing a low-cost, highly efficient protein purification system.

Lipoate-protein ligase A (LplA), which is responsible for lipoylation in *E. coli*, is a well-folded, monomeric globular protein with a molecular weight of 38 kDa. In vitro, it exhibits structural self-assembly and lower critical solution temperature (LCST)-type reversible phase separation, and is capable of forming controllable orthogonal condensates [22]. In this study, we applied LplA to the field of protein purification. We constructed a universal vector for the fusion expression of LplA and the target protein, developed a protein purification method based on condensates, and validated the method using GFP, demonstrating that high-purity protein can be obtained after just a single phase transition cycle. In addition, enzymes of various molecular sizes were employed to test the versatility of this method. And this method was compared with traditional affinity chromatography. In summary, our study presents a novel condensate-based protein purification method that can be applied to the purification of many other proteins.

## 2. Results

### 2.1. Expression and Purification of LplA

**Figure 1:**
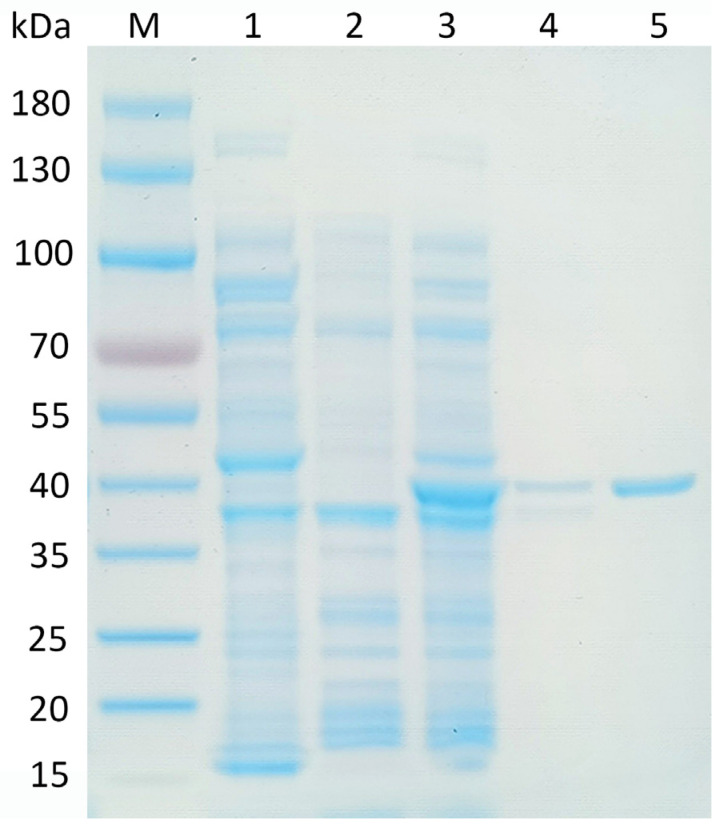
SDS-PAGE analysis of LplA: lane M: standard marker proteins; lane 1: supernatant of cell lysate (BL21-pET28a); lane 2: precipitation of cell lysate (BL21-pET28a); lane 3: supernatant of cell lysate (BL21-pET28a-LplA); lane 4: precipitation of cell lysate (BL21-pET28a-LplA); lane 5: the purified LplA protein.

The recombinant plasmid (pET28a-LplA) was transformed into *E. coli* BL21 (DE3) cells, and the recombinant strain (BL21-pET28a-LplA) was grown in LB medium containing 50 μg/mL kanamycin. After culturing at 37 °C for 1.5 h until the OD_600_ reached 0.5–0.6, IPTG was added to a final concentration of 0.3 mM, and then expression was induced at 16 °C for 16 h. LplA was overexpressed in the form of a soluble protein (Figure 1). After ultrasonic disruption of the cells, the supernatant of the lysate was incubated at 30 °C for 30 min. The resulting condensates were collected by centrifugation and subsequently dissolved at 4 °C to obtain the purified protein. As shown in Figure 1, the purified LplA migrated as a single band on SDS–PAGE with an apparent molecular weight of approximately 37.9 kDa, consistent with the calculated value. These results demonstrate that LplA can form condensates and separate from solution upon temperature elevation, indicating its potential as a temperature-sensitive tag for protein purification.

### 2.2. Selection of LLPS Temperature and Purification of Fusion Protein

**Figure 2:**
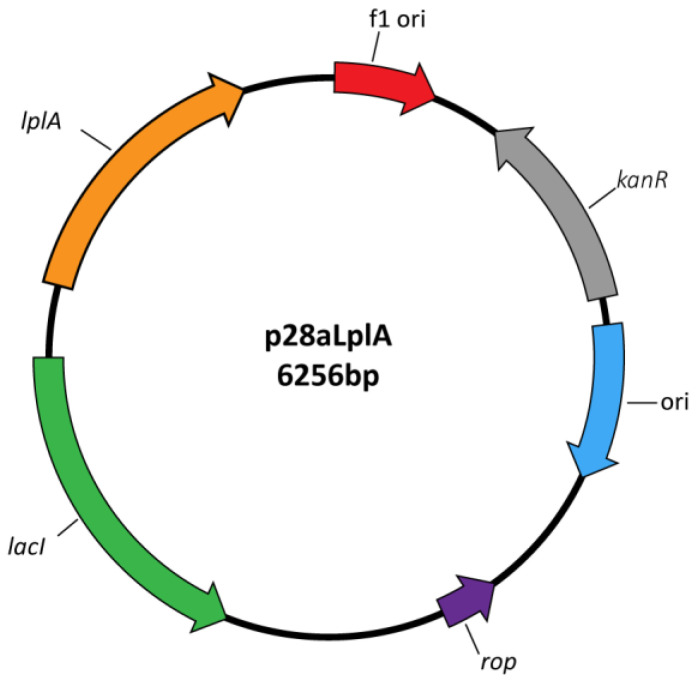
The vector map of p28aLplA.

**Figure 3:**
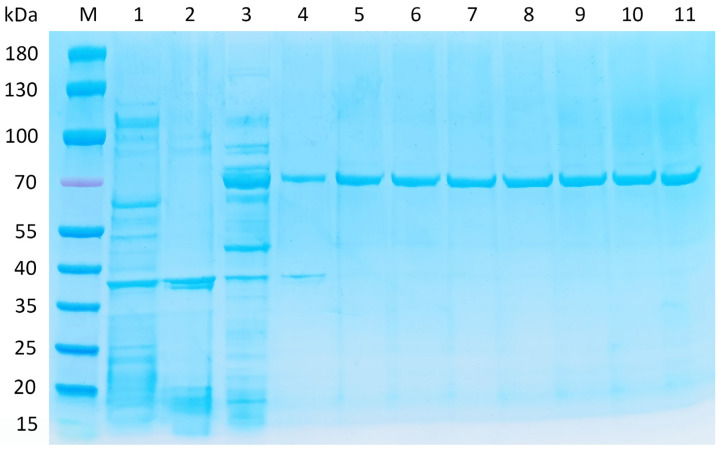
SDS-PAGE analysis of the fusion protein (LplA-GFP): lane M: standard marker proteins; lane 1: supernatant of cell lysate (BL21-pET28a); lane 2: precipitation of cell lysate (BL21-pET28a); lane 3: supernatant of cell lysate (BL21-p28aLplA-GFP); lane 4: precipitation of cell lysate (BL21- p28aLplA-GFP); lane 5: the purified LplA-GFP protein (formed condensates at 20 °C); lane 6: the purified LplA-GFP protein (formed condensates at 23 °C); lane 7: the purified LplA-GFP protein (formed condensates at 25 °C); lane 8: the purified LplA-GFP protein (formed condensates at 27 °C); lane 9: the purified LplA-GFP protein (formed condensates at 30 °C); lane 10: the purified LplA-GFP protein (formed condensates at 32 °C); lane 11: the purified LplA-GFP protein (formed condensates at 35 °C).

As the LplA protein forms condensates upon heating and separates from the solution, the universal vector p28aLplA was constructed for the expression of a fusion protein using the *lplA* gene (Figure 2). In addition, the temperature conditions required for condensate formation during fusion protein purification were explored by subjecting the fusion protein LplA-GFP to LLPS at 20 °C, 23 °C, 25 °C, 27 °C, 30 °C, 32 °C, and 35 °C. Figure 3 shows that LplA-GFP was efficiently expressed in *E. coli* and predominantly existed in a soluble form. Following purification by temperature modulation, SDS–PAGE analysis revealed a distinct single band corresponding to a molecular weight of approximately 68 kDa, consistent with the expected molecular weight of LplA-GFP. These results indicate that the fusion protein LplA-GFP was successfully purified from the cell lysate through temperature-induced phase separation.

**Figure 4:**
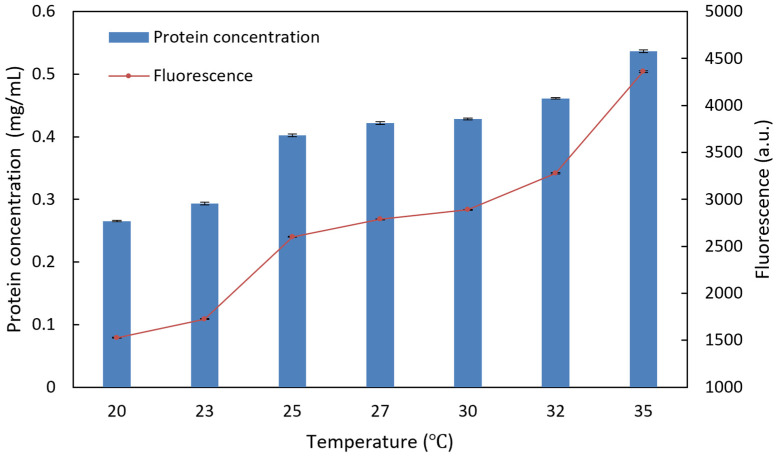
The concentration and fluorescence intensity of the purified LplA-GFP protein obtained by phase separation at different temperatures.

Furthermore, as shown in Figure 4, within the temperature range of 20–35 °C, the purification efficiency of LplA-GFP gradually increased as the LLPS temperature rose, and the GFP exhibited good activity. Taking into account both purification efficiency and protein stability, 30 °C was adopted as the LLPS temperature for all subsequent experiments.

**Figure 5:**
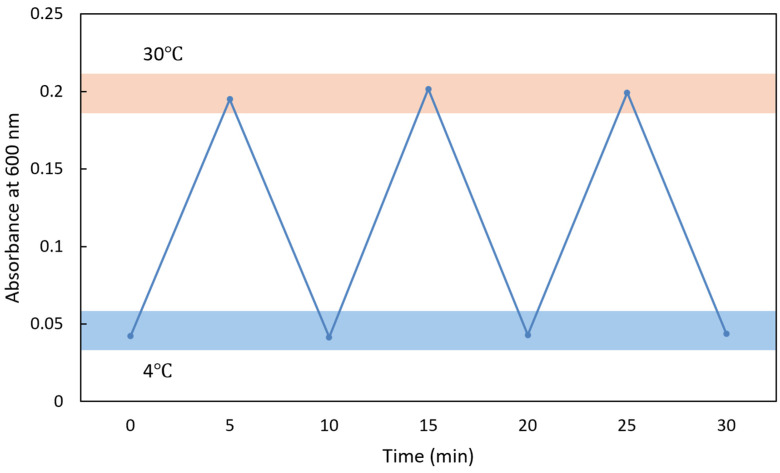
The solution-condensate reversible cycle of LplA-GFP characterized using turbidity at 600 nm.

To verify that the fusion protein undergoes phase separation to form condensates under elevated temperature rather than forming aggregate precipitates, a temperature-cycling solution-condensate reversibility experiment was conducted on LplA-GFP, with condensate formation assessed by measuring absorbance at 600 nm. Three cycles were performed between 4 °C and 30 °C. The solution’s absorbance changed rapidly with temperature, demonstrating good reversibility during the solution-condensate cycling, and no insoluble particles or precipitates were observed during the temperature cycling (Figure 5). This indicates that the fusion protein also exhibits LCST behaviour, forming condensates rather than precipitates under heating conditions.

### 2.3. The Versatility of LplA as a Purification Tag

**Figure 6:**
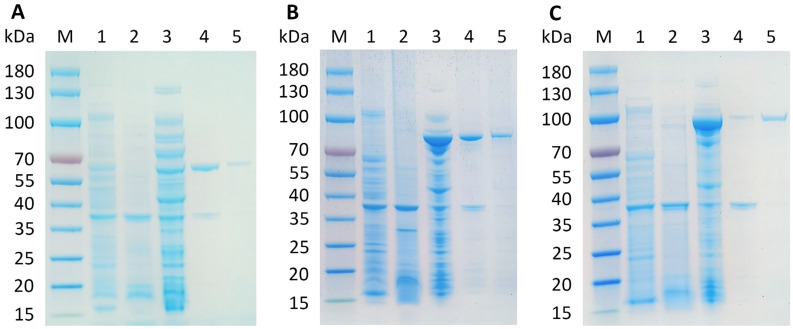
SDS-PAGE analysis of LplA-EstA, LplA-BcsZ, and LplA-EglS: (**A**) lane M: standard marker proteins; lane 1: supernatant of cell lysate (BL21-pET28a); lane 2: precipitation of cell lysate (BL21-pET28a); lane 3: supernatant of cell lysate (BL21-p28aLplA-estA); lane 4: precipitation of cell lysate (BL21-p28aLplA-estA); lane 5: the purified LplA-EstA protein. (**B**) lane M: standard marker proteins; lane 1: supernatant of cell lysate (BL21-pET28a); lane 2: precipitation of cell lysate (BL21-pET28a); lane 3: supernatant of cell lysate (BL21-p28aLplA-bcsZ); lane 4: precipitation of cell lysate (BL21-p28aLplA-bcsZ); lane 5: the purified LplA-BcsZ protein. (**C**) lane M: standard marker proteins; lane 1: supernatant of cell lysate (BL21-pET28a); lane 2: precipitation of cell lysate (BL21-pET28a); lane 3: supernatant of cell lysate (BL21-p28aLplA-eglS); lane 4: precipitation of cell lysate (BL21-p28aLplA-eglS); lane 5: the purified LplA-EglS protein.

To expand the applications of this purification method, three enzymes of different molecular sizes (lipase gene *estA* 546 bp, endoglucanase gene *bcsZ* 1041 bp, and the endoglucanase gene *eglS* 1413 bp) were selected as target proteins. SDS-PAGE analysis showed that the fusion proteins LplA-EstA, LplA-BcsZ, and LplA-EglS were all successfully expressed in *E. coli* BL21 (DE3). The fusion proteins formed condensates and separated from the solution at 30 °C, and were dissolved in PBS buffer at 4 °C. The SDS-PAGE results showed that the fusion protein had been successfully purified, appearing as single bands with molecular weights of approximately 59 kDa, 79 kDa, and 92 kDa, respectively, which corresponded to the expected molecular weights (Figure 6).

### 2.4. Comparison Between Condensate-Based Purification and Ni-NTA Affinity Chromatography

The study compared condensate-based purification with conventional Ni-NTA affinity chromatography and evaluated the activities of the enzymes purified using these two methods. The recombinant plasmids (pET28a-estA, pET28a-bcsZ, pET28a-eglS) were transformed into *E. coli* BL21 (DE3) cells, which were then induced with 0.3 mM IPTG at 16 °C for 16 h. The free enzymes (without LplA) EstA, BscZ, and EglS were purified by Ni-NTA affinity chromatography, followed by ultrafiltration to remove imidazole. Table 1 presents the measured specific activities. Among them, the specific activity and purification fold of the lipase EstA purified using the LplA tag were significantly higher than those of the free enzyme purified by Ni-NTA affinity chromatography. The specific activities and purification folds of the endoglucanases BcsZ and EglS purified using the LplA tag were slightly lower than those of the corresponding free enzymes purified by Ni-NTA affinity chromatography. However, they still retained relatively high specific activities, indicating that the LplA tag did not significantly impair the catalytic functions of the enzymes. The reduction in specific activity may be attributed to the relatively large molecular weight of LplA, which could hinder substrate access to the active pocket. Enzymatic activity could subsequently be restored through the removal of the LplA tag. The higher specific activity of LplA-EstA compared with free EstA may be attributed to the fact that LplA does not prevent substrate access to the enzyme’s active site, and the mild conditions of the condensate-based purification method effectively preserve enzyme activity.

In addition, the purity, yield, and recovery rate of the proteins obtained using these two purification methods were measured (Table 2). The results showed that the proteins purified using the LplA tag and Ni-NTA affinity chromatography had similar purity levels, while the LplA tag-based purification method provided a higher protein yield and recovery rate.

## 3. Discussion

Protein condensates are dynamic membraneless assemblies formed through multivalent protein–protein interactions and are typically characterized by reversibility and responsiveness to environmental factors such as temperature, pH, and ionic strength [23]. Recently, biomolecular condensates have been considered capable of enabling numerous potential applications across different fields. For example, Schneider et al. utilized condensates to enhance transcriptional activation in cells [24], whereas Shapiro et al. employed condensates to promote mRNA translation in *E. coli*, thereby improving protein expression [25]. In this study, for the first time, proteins capable of forming condensates were utilized as temperature-sensitive purification tags for the purification of other proteins.

Protein purification is a crucial step in biochemical, structural biological, and bioengineering research. High-purity proteins are of great significance for enzymatic analysis, structural determination, and functional research [26]. At present, protein purification mainly relies on methods such as affinity chromatography [27], ion-exchange chromatography [28], and size-exclusion chromatography [29]. Although these methods exhibit high purification efficiency and broad applicability, they generally require expensive chromatographic resins and equipment and involve relatively complex purification processes with multiple buffer exchange and elution steps. These requirements not only increase experimental costs and operation time, but also lead to target protein loss or reduced biological activity [30,31]. In addition, chromatographic resins also face challenges such as resin inactivation and limited reusability in large-scale applications. Here, we have established a novel one-step purification method based on protein condensates. This method is simple, requires a short processing time, and enables the separation of the target protein under mild conditions, thereby significantly improving experimental and production efficiency while helping to preserve the target protein’s native conformation and biological activity.

In this study, the condensate-forming protein LplA was utilized as a purification tag. It was first demonstrated that LplA could form condensates and separate from solution upon temperature elevation, and subsequently redissolve in buffer after cooling, while maintaining a high degree of protein purity. The universal vector p28aLplA for the fusion expression of LplA and target proteins was then constructed, and the purification strategy was validated using GFP as a reporter protein. An appropriate temperature is critical for condensate formation. In this study, phase separation of the fusion proteins was performed at 20 °C, 23 °C, 25 °C, 27 °C, 30 °C, 32 °C, and 35 °C. The results showed that the purification efficiency increased with increasing temperature. Considering both purification efficiency and protein activity retention, 30 °C was selected as the optimal phase-separation temperature for subsequent experiments.

Versatility is of great importance for the development of novel purification strategies. In this study, three enzymes with different molecular sizes were successfully purified, demonstrating the practicality and broad applicability of this condensate-based purification method. The specific activities of the purified enzymes were evaluated and compared with those of the corresponding free enzymes purified by Ni-NTA affinity chromatography. The results showed that the specific activities were generally comparable, while the LplA-EstA exhibited significantly higher specific activity than the free enzyme purified by Ni–NTA affinity chromatography. We speculated that the slightly reduced specific activities of the endoglucanases may be attributed to the relatively large molecular weight of LplA, which could hinder substrate access to the active pocket. In future studies, we plan to restore enzymatic activity by removing the LplA tag. Finally, the purity, yield, and recovery rate of the purified proteins were measured. The results showed that proteins purified using both methods exhibited high purity, but the condensate-based purification method performed better in terms of yield and recovery rate. Therefore, the condensate-based purification strategy exhibits clear advantages in preserving protein activity and purification efficiency. Nevertheless, owing to the dynamic nature of proteins, the purification performance of the LplA tag may vary among different target proteins and expression systems, and this requires further validation in subsequent studies.

## 4. Materials and Methods

### 4.1. Bacterial Strains and Plasmids

The gene encoding the *E. coli* LplA protein (GenBank Accession No. ACT46042.1) used in this study was amplified by PCR using the genomic DNA of the laboratory-preserved *E. coli* BL21(DE3) strain as the template. *E. coli* DH5α (Weidi, Shanghai, China) was used for plasmid construction and routine propagation, whereas *E. coli* BL21(DE3) (Weidi, Shanghai, China) was used for protein expression. The plasmid pET-28a(+) was preserved in our laboratory, where it was built from scratch, and served as a plasmid backbone for the construction of protein expression vectors. The genes encoding the lipase EstA (GenBank Accession No. NP_388152.1), the endoglucanase BcsZ (GenBank Accession No. ACT45180.1), and the β-1,4-endoglucanase EglS (GenBank Accession No. Z29076.1) were synthesized by GenScript (Nanjing, China) without signal peptide sequences. The green fluorescent protein GFP gene (GenBank Accession No. KF410615.1) was preserved in our laboratory.

### 4.2. Chemicals

Phanta Flash Master Mix (Dye Plus) and Rapid Taq Master Mix were purchased from Vazyme (Nanjing, China). The HB-infusion Seamless Cloning Kit was obtained from Hanbio (Shanghai, China). DpnI was purchased from New England Biolabs (Beijing, China). *p*-nitrophenol and 4-nitrophenyl butyrate were purchased from Aladdin (Shanghai, China). PBS buffer was purchased from Cytiva (Shanghai, China). Other molecular biology reagents and chemical reagents of analytical grade were commercially available.

### 4.3. Cloning and Expression of LplA in E. coli

Using the *E. coli* BL21 (DE3) genome and the pET-28a(+) plasmid as templates, the *lplA* gene and the pET28a plasmid backbone were amplified using the primer pairs LplA-F/LplA-R1 and pET28a-F1/pET28a-R1, respectively (Table 3). The *lplA* fragment was then seamlessly cloned into the pET28a backbone to construct the recombinant plasmid pET28a-LplA [32]. Positive clones were selected and verified by DNA sequencing, after which the recombinant plasmid pET28a-LplA was transformed into *E. coli* BL21(DE3) [33].

The recombinant cells (BL21-pET28a-LplA) were cultured at 37 °C until the optical density (600 nm) reached 0.6. LplA expression was induced by the addition of IPTG to a final concentration of 0.3 mM, followed by incubation at 16 °C for 16 h. The cells were then harvested and washed with PBS buffer (137 mM NaCl, 2.7 mM KCl, 10 mM Na_2_HPO_4_, and 1.8 mM KH_2_PO_4_) [34]. The collected cells were resuspended in PBS buffer and disrupted by ultrasonication at 220 W for 20 min [35]. The cell lysate was centrifuged at 4 °C, and the supernatant was collected.

### 4.4. Condensation Validation of LplA

The supernatant of the BL21-pET28a-LplA cell lysate was incubated at 30 °C for 30 min, followed by centrifugation at 10,000 rpm for 20 min to collect the condensates. The condensates were washed twice with PBS buffer to remove contaminant proteins. Subsequently, the condensates were redissolved in an equal volume of PBS buffer at 4 °C, and insoluble proteins were removed by centrifugation at 12,000 rpm for 20 min at 4 °C. The resulting supernatant was collected as the purified LplA protein. The purified protein was analysed by sodium dodecyl sulfate–polyacrylamide gel electrophoresis (SDS–PAGE).

### 4.5. Construction of a Universal Plasmid

A universal vector for the fusion expression of the condensate-forming protein and target proteins was constructed. pET-28a (+) was used as the backbone. The *lplA* gene fragment and the pET28a plasmid backbone were amplified using the primer pairs LplA-F/LplA-R2 and pET28a-F2/pET28a-R2, respectively (Table 3). Following seamless cloning, the universal vector p28aLplA was constructed and subsequently verified by DNA sequencing.

### 4.6. LLPS Temperature Setting and Condensate Purification of GFP

To explore the optimal purification conditions, the LLPS temperature was investigated. Phase separation of the fusion proteins was performed at 20 °C, 23 °C, 25 °C, 27 °C, 30 °C, 32 °C, and 35 °C. Green fluorescent protein (GFP) was selected as a reporter protein to evaluate the effects of different temperatures on purification efficiency. A thrombin cleavage site was inserted between LplA and GFP to enable separation of LplA from the target protein when necessary.

For plasmid construction, the *gfp* gene containing a thrombin cleavage site sequence was first amplified using the primer pair GFP-F/GFP-R carrying the thrombin cleavage site sequence. The p28aLplA backbone was amplified using the primer pair p28aLplA-F/p28aLplA-R (Table 3). The amplified fragments were then assembled by seamless cloning to generate the recombinant plasmid p28aLplA-GFP.

The recombinant plasmid p28aLplA-GFP was transformed into *E. coli* BL21 (DE3), while plasmid pET28a was expressed in *E. coli* BL21 (DE3) as a control. The transformants were induced with 0.3 mM IPTG at 16 °C for 16 h. The cells were harvested, washed twice with PBS buffer, and disrupted by ultrasonication. The fusion protein was purified by the formation of condensates under different temperature conditions, using the same method as above. Subsequently, SDS-PAGE analysis was performed on the crude extract and the purified fusion protein, and the concentration of the purified fusion protein was determined using the Bradford method [36]. At the same time, 200 μL of the purified protein solution obtained at different temperatures was transferred to a 96-well black opaque microplate. Measurements were performed on a FlexStation 3 multi-function microplate reader (Shanghai, China) with excitation at 495 nm and emission at 525 nm [37]. The GFP signal was recorded before processing in Microsoft Excel.

### 4.7. Purification of Lipase and Endoglucanase

To evaluate the versatility of this purification method, lipase (EstA) and endoglucanases (BscZ, EglS) of different molecular sizes were selected as target proteins. The plasmids p28aLplA-estA, p28aLplA-bcsZ, and p28aLplA-eglS were synthesised at GenScript (Nanjing, China). The target protein genes were located at the 3′ end of the *lplA* gene, with a thrombin cleavage site sequence inserted between the target protein gene and *lplA*.

At the same time, the plasmids p28aLplA-estA, p28aLplA-bcsZ, and p28aLplA-eglS were reverse-amplified using primers p28estA-F, p28bcsZ-F, p28eglS-F, and pET28a-R3, respectively (Table 3). These PCR products were digested with Dpn I and transformed into *E. coli* DH5α for cyclisation. Following cultivation, the plasmids were extracted, resulting in pET28a-estA, pET28a-bcsZ, and pET28a-eglS as controls.

**Figure 7:**
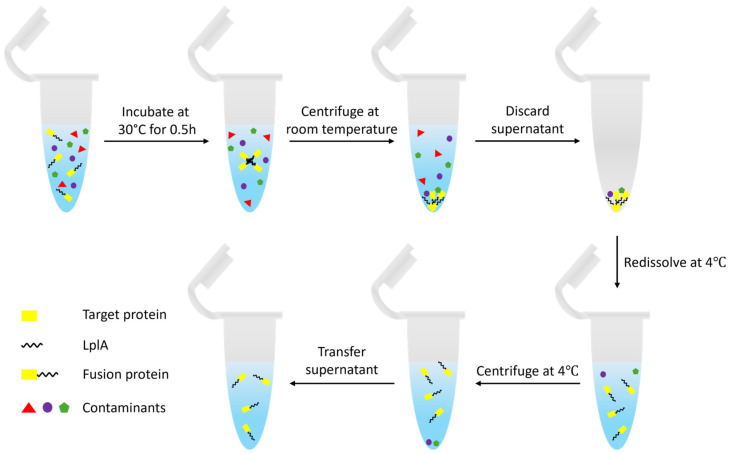
The flowchart of a protein purification method based on condensates.

The recombinant plasmids were individually transformed into *E. coli* BL21(DE3). Expression was induced with 0.3 mM IPTG at 16 °C for 16 h. Cells were harvested by centrifugation, washed twice with PBS buffer, and disrupted by ultrasonication. Fusion proteins (LplA-EstA/BcsZ/EglS) were purified via temperature-induced LLPS through condensate formation, following the method described above (Figure 7). The crude extract and the purified fusion protein were then analysed by SDS-PAGE. The free enzyme (without LplA) was purified using Ni Sepharose excel (Cytiva, Shanghai, China) in accordance with the manufacturer’s instructions [38]. The protein concentrations of the purified fusion protein and the free enzyme were measured via the Bradford method. The protein purity was calculated by ImageJ (Version 1.54p) grayscale analysis. The yield was calculated by dividing the mass of purified target protein by the culture volume.

### 4.8. Activity Detection of Lipase and Endoglucanase

Lipase activity was determined using the *p*-nitrophenyl (pNP) colorimetric assay with 4-nitrophenyl butyrate (pNPB) as the substrate [39]. Briefly, 10.46 mg of pNPB was dissolved in 1 mL isopropanol to obtain an approximately 50 mM stock solution. The pNPB stock solution was then diluted to 5 mM using 9 mL of 50 mM Tris-HCl buffer (pH 8.0). The reaction system consisted of 160 μL Tris-HCl buffer, 20 μL pNPB substrate, and 20 μL enzyme solution. The reaction was incubated at 37 °C for 10 min and subsequently terminated by adding 50 μL of 1 M Na_2_CO_3_. The absorbance of the reaction mixture was measured at 405 nm using a spectrophotometer. One unit of lipase activity corresponds to the catalytic production of 1 μmol of pNP per minute under standard conditions.

Endoglucanase activity was determined using the Endo-β-1,4-glucanase/cellulase activity assay kit (Grace, Suzhou, China) in accordance with the manufacturer’s instructions [40]. One unit of endoglucanase activity corresponds to the catalytic production of 1 μg of reducing sugar per hour under standard conditions.

## 5. Conclusions

In summary, we applied condensate proteins to the field of protein purification for the first time; developed a temperature-sensitive protein purification tag, LplA; and established a new one-step protein purification method. A universal vector was constructed for the fusion expression of LplA and the target protein. Additionally, 30 °C was identified as the optimal temperature for LLPS purification. The practicality of this condensate-based purification method was verified using proteins of several different molecular sizes. Finally, a comparison of enzyme activity and purification efficiency with those of the free enzymes purified by Ni-NTA affinity chromatography demonstrated that this purification method effectively preserved the protein’s biological activity and achieved high purification efficiency. The condensate-based protein purification method is simple to operate, requires a short purification cycle, offers high purification efficiency, and employs mild conditions that effectively preserve protein activity and function, making it suitable for the production and purification of functional proteins. This study represents a valuable endeavour and provides new avenues for research into the application of condensate proteins in protein purification.

## Figures and Tables

**Table 1:** Enzyme activity of lipase and endoglucanases. LplA enzymes were purified by condensate. Free enzymes were purified by Ni-NTA affinity chromatography.

Sample	Specific Activity (U/mg)	Purification (Fold)
LplA-EstA crude extract	0.024 ± 0.001	1.000
LplA-EstA	3.214 ± 0.097 ***	133.917
Free EstA	2.398 ± 0.058	99.917
LplA-BcsZ crude extract	13.47 ± 0.26	1.00
LplA-BcsZ	487.32 ± 13.92 *	36.18
Free BcsZ	545.47 ± 15.76	40.49
LplA-EglS crude extract	48.94 ± 3.03	1.00
LplA-EglS	3036.13 ± 29.57 *	62.04
Free EglS	3156.11 ± 30.14	64.49

Data are expressed as the mean ± s.d. for *n* = 3. Significance was assessed by *t*-test between LplA enzyme and the corresponding free enzyme. * *p* < 0.05, *** *p* < 0.0005.

**Table 2:** The purity, yield, and recovery rate of purified proteins. LplA enzymes were purified by condensate. Free enzymes were purified by Ni-NTA affinity chromatography.

Sample	Purity (%)	Yield (mg/L)	Recovery Rate (%)
LplA-estA	95.32	18.10	63.13
Free-estA	94.62	16.95	51.60
LplA-bcsZ	93.75	30.15	66.74
Free-bcsZ	91.27	17.75	53.62
LplA-eglS	94.08	20.80	75.56
Free-eglS	95.31	17.40	61.33

**Table 3:** Primers used in this study.

Designation	Primer Sequence (5′-3′)
LplA-F	ATGTCCACATTACGCCTGCT
LplA-R1	GATCTCATTACCTTACCGCCCCCG
LplA-R2	GCTGCCGCGCGGCACCAGCCTTACCGCCCCCGC
pET28a-F1	GGGCGGTAAGGTAATGAGATCCGGCTGCTAACAA
pET28a-F2	GTGCCGCGCGGCAGCTGAGATCCGGCTGCTAACAA
pET28a-R1	GCGTAATGTGGACATGCGACCCATTTGCTGTCC
pET28a-R2	GCGTAATGTGGACATGTGATGATGATGATGATGGCTGC
pET28a-R3	GTGATGATGATGATGATGGCTGC
GFP-F	ATGCGTAAAGGCGAGGAACT
GFP-R	TTATTTATACAGTTCATCCATACCGTGGGT
p28aLplA-F	GAACTGTATAAATAAGATCCGGCTGCTAACAAAGC
p28aLplA-R	CTCGCCTTTACGCATGCTGCCGCGCGG
p28estA-F	CATCATCATCATCACGCAGAGCACAACCCGG
p28bcsZ-F	CATCATCATCATCACTGTACCTGGCCTGCCTGG
p28eglS-F	CATCATCATCATCACGCTGGTACGAAAACCCCG

## Data Availability

The original contributions presented in this study are included in the article. Further inquiries can be directed to the corresponding author.
